# Policy Guidance for Direct-to-Consumer Genetic Testing Services: Framework Development Study

**DOI:** 10.2196/47389

**Published:** 2024-07-17

**Authors:** Suzanne Maria Onstwedder, Marleen Elizabeth Jansen, Martina Cornelia Cornel, Tessel Rigter

**Affiliations:** 1 Department of Public Health Genomics and Screening Centre for Health Protection Dutch National Institute for Public Health and the Environment Bilthoven Netherlands; 2 Section Community Genetics Department of Human Genetics Amsterdam UMC, Vrije Universiteit Amsterdam Amsterdam Netherlands; 3 Personalized Medicine Programme Amsterdam Public Health Research Institute Amsterdam Netherlands

**Keywords:** genetic testing, direct-to-consumer testing, health policy, genetic privacy, online market, informed consent, public health genomics, policy decision, mHealth, mobile health, privacy

## Abstract

**Background:**

The online offer of commercial genetic tests, also called direct-to-consumer genetic tests (DTC-GTs), enables citizens to gain insight into their health and disease risk based on their genetic profiles. DTC-GT offers often consist of a combination of services or aspects, including advertisements, information, DNA analysis, and medical or lifestyle advice. The risks and benefits of DTC-GT services have been debated and studied extensively, but instruments that assess DTC-GT services and aid policy are lacking. This leads to uncertainty among policy makers, law enforcers, and regulators on how to ensure and balance both public safety and autonomy and about the responsibilities these 3 parties have toward the public.

**Objective:**

This study aimed to develop a framework that outlines aspects of DTC-GTs that lead to policy issues and to help provide policy guidance regarding DTC-GT services.

**Methods:**

We performed 3 steps: (1) an integrative literature review to identify risks and benefits of DTC-GT services for consumers and society in Embase and Medline (January 2014-June 2022), (2) structuring benefits and risks in different steps of the consumer journey, and (3) development of a checklist for policy guidance.

**Results:**

Potential risks and benefits of DTC-GT services were mapped from 134 papers and structured into 6 phases. In summary, these phases were called the consumer journey: (1) exposure, (2) pretest information, (3) DNA analysis, (4) data management, (5) posttest information, and (6) individual and societal impact. The checklist for evaluation of DTC-GT services consisted of 8 themes, covering 38 items that may raise policy issues in DTC-GT services. The themes included the following aspects: general service content, validity and quality assurance, potential data and privacy risks, scientific evidence and robustness, and quality of the provided information.

**Conclusions:**

Both the consumer journey and the checklist break the DTC-GT offer down into key aspects that may impact and compromise individual and public health, safety, and autonomy. This framework helps policy makers, regulators, and law enforcers develop methods to interpret, assess, and act in the DTC-GT service market.

## Introduction

### Background

Since the development and marketing of commercial genetic tests, citizens have been able to buy DNA tests from the internet without any direct involvement of a medical professional [1]. Using these DNA tests, consumers—here viewed as individuals who have purchased and taken a commercial genetic test or potential buyers who are exposed to these offers—are now able to gain insight into their health characteristics and risks of disease. These digital health consumer products are an example of those technological innovations that the private sector has picked up from research. Their availability fits with the trends of the commercial market, which reflect the consumerization of mobile health (mHealth) worldwide in numerous areas of health care, fitness, and wellness. The rise of the consumer DNA test market has mainly been fueled by a drop in the cost of collecting and analyzing genomic data [2]. Meanwhile, the public is increasingly showing an interest in genomics [3].

These DNA tests are also called direct-to-consumer genetic tests (DTC-GTs) and, at a minimum, entail a service to collect a saliva sample (sending a kit, giving instructions on how to collect and return the sample), conduct a genetic test of the sample, and provide a report of outcomes of the test. Because the DTC-GT process entails multiple aspects, in this study, we described them as “DTC-GT services.” There are many types of DTC-GT services, including tests for ancestry and paternity. This paper focused on DTC-GT services aimed at health. Additional digital services are often provided by means of personal health information and lifestyle advice in apps or reports, online consultation with genetic experts, and privacy-ensured storage of DNA data [4]. In this way, DTC-GT services combine aspects of many mHealth products that assess and monitor health. After purchase, consumers are often able to download their raw genetic data and explore the possibilities of multiple online third-party analyzers that will run an additional analysis on their data [5]. In this way, these third-party analyzers further connect DTC-GT users to the scientific literature and promise to make novel research insights accessible to users [6]. This illustrates that DTC-GTs are part of a complex international online market [5,6].

DTC-GT companies generally state that their services can inform health decisions and minimize disease risk by offering insight into personal health information and providing lifestyle advice [7,8]. Although these statements sound promising in relation to health improvement, their validity and utility in this field is under debate [9]. There is also further uncertainty about issues such as privacy, psychological impact, and the effects on health care systems [10].

Consequently, it can be argued that the risks and benefits of DTC-GTs are valid reasons for developing policies on this topic. However, ensuring that these issues are addressed at the right level is challenging. The fact that these tests are offered internationally via the web complicates any effort to address their potential risks or benefits. Some of these issues are regulated at European or international levels, while others are regulated at national or regional levels [11]. Addressing these issues raises questions regarding the roles and responsibilities of policy makers, regulators, and law enforcers, both within and outside the health care system. Insight is needed into the potential risks and benefits of DTC-GT services that would lead to the development of policies. A structured overview of potential risks and benefits could guide policy on DTC-GT services. Such an overview would enable policy makers, regulators, and law enforcers to identify their responsibilities. For example, policy makers at the national government level, professional associations, grant providers, and patient and consumer organizations would then be able to define the outstanding questions for research and needs for publicly available information to ultimately enable responsible availability and use of DTC-GTs.

### Risks and Benefits of DTC-GT Services

Genomics holds the potential to improve diagnosis, personalize treatment, and determine groups at risk of developing a disease. Researchers are continuously extending their knowledge of genomics and its potential impact on individual health, personalized medicine, prevention [12-14], public health, and precision public health [15]. It is argued that gaining insight into personal health risks, which are impacted by genetic risks, lifestyle factors, and environmental effects, could ultimately help empower citizens to gain more personal control over their health. Having said that, it seems promising that consumers can autonomously impact their health through DTC-GT services. Yet, genetic and ethics experts have expressed their concerns about DTC-GTs [9,10]. Experts argue that the information provided can be misleading and that results that indicate an increased or reduced disease risk can easily be misinterpreted, potentially leading to misguided health decisions and medical risks [16,17]. This leads to the question of whether DTC-GT services are an effective and safe way for the public to pursue health improvement.

However, it could be argued that DTC-GT services should not be judged solely as a medical product and that arguments about personal utility and autonomy should also be part of the equation [18-21]. One could make the case that a consumer product may serve a purpose other than a medical one, an educational one, for instance. Therefore, the risks and benefits of DTC-GT services as a consumer product could be weighed differently. When purchasing a test, aspects of a product, such as ease of use and accessibility, may be a stronger factor in consumer decision-making, than the test being 100% accurate.

### Policy and Regulatory Issues

Due to a lack of consensus about the net benefit or risk of DTC-GTs, uncertainty has arisen among policy makers, regulators, and law enforcers about their roles and responsibilities in the DTC-GT market. DTC-GT services fall into a gap of regulatory structure in both Europe and the United States [11,22]. Currently, no specific DTC-GT legislative instruments are being implemented in most of the member states of Europe or in the United States. A variety of laws, all on different levels of legislation (state or country specific, European, and international laws), are effective in the DTC-GT market. These laws cover aspects such as medical device safety, laboratory quality assurance, medical supervision, genetic counseling, and informed consent [11,22-24]. Yet, most of these laws only apply to genetic testing within the conventional health care system. Applying these laws to a commercial product may not be sufficient to protect citizens from potentially harmful tests.

Further regulatory difficulties are caused by the international and dynamic character of DTC-GT services. A great variety of tests for health and other purposes are available online. Examples include cancer risk tests, nutrigenetic testing, and ancestry testing, combined with insights into health characteristics and other traits [4,22]. Moreover, the additional services that are offered, such as apps or reports containing personal health and lifestyle advice, add further variety to their offers [4]. Consumers can purchase any of these DTC-GT services on the web from many places in the world. The global and dynamic nature of the market hinders regulatory bodies in implementing effective policies and in law enforcement.

### Ambiguity Calls for Policy Guidance

The ambiguity in the legislative framework applicable to DTC-GTs has resulted in challenges for policy makers, regulators, and law enforcers in protecting citizens from potentially harmful services. Meanwhile, the impact of DTC-GTs on citizens and their health continues to be a topic of debate. To balance safety and individual autonomy, all aspects of DTC-GT services should be considered when regulatory decisions are required [11]. For this reason, it is important that the current body of knowledge on the risks and benefits of all key aspects of DTC-GTs is translated into policy guidance. To ensure effective policy making, researchers have found the following issues to be relevant in regulating the DTC-GT market: autonomy and welfarism, informed decision-making, privacy, clinical validity and utility, perspectives of the public and health care professionals, the role of regulatory organizations, legislation on genetic testing, and laws protecting against genetic discrimination [25]. All these issues are interlinked in DTC-GT services. The existing literature has focused on gaining insight into these issues and has started to collect empirical evidence on the impact of the market. Yet, instruments that assess those aspects of DTC-GT services that are likely to need regulation are lacking.

To ensure that policy guidance covers all aspects of DTC-GT services, it is important to include the risks and benefits of each distinct service element offered by commercial companies. To this end, in this study, we described those aspects of DTC-GT services that may give rise to policy issues. Subsequently, we synthesized a framework for policy makers, law enforcers, and regulators to develop methods to interpret, assess, and act in the DTC-GT service market.

The framework development process consisted of 3 steps:

- Performing an integrative literature review to identify the risks and benefits of DTC-GT services to consumers and society
- Structuring the benefits and risks of each step of the consumer journey
- Developing a checklist for policy guidance

## Methods

### Study Design

This study focused on DTC-GT services that advertise that they deliver insight into disease risks and lifestyles that promise to impact customer health through behavior change. This includes anticipated lifestyle changes, changes to medication intake, and recommendations to seek medical help from professionals. We viewed consumers of the DTC-GT market as both individuals who have purchased DTC-GT services and as potential consumers who are exposed to offers of these services. These services are offered via webshops, including primary DTC-GT service companies and secondary selling points, such as Amazon, and at high street stores.

### Study Layout

To distill key aspects of DTC-GTs, those services that lead to policy issues, potential risks, and benefits for consumers and society, including and beyond medical implications, were systematically mapped by means of an integrative literature review (step 1). To do this, the complete DTC-GT service pathway was studied, from exposure to the offer of DTC-GTs to the ultimate individual and societal impacts of DTC-GT services.

In step 2, the risks and benefits were structured into phases, following each step the consumer takes along the DTC-GT service pathway. Each of these phases triggered distinct policy issues, including issues regarding informed decision-making, privacy, clinical validity and utility, and legislation for genetic testing [25].

Step 3 involved developing a checklist that can ultimately offer guidance on each distinct DTC-GT policy issue. To ensure adequate policy guidance, checklist items that help evaluate all phases of the DTC-GT service pathway were included. Every checklist item reflects a potential risk or benefit found in the literature review.

### Study Identification and Eligibility Check

The risks and opportunities associated with DTC-GTs were determined by means of a literature search that focused on health, including lifestyle and disease risk. The literature search was performed systematically using Embase.com, which combines the databases of Embase and Medline. The search strategy included the following primary search terms with corresponding synonyms: “direct-to-consumer genetic testing,” “impact,” “risks,” “opportunities,” “clinical study,” “evidence based practice,” “accuracy,” and “ethical, legal, and social issues” (see Multimedia Appendix 1). In addition, 5 experts in the field of genomics, public health, and biomedical sciences with knowledge of DTC-GTs were asked to provide key papers discussing the risks and benefits of DTC-GTs.

Publications were included if they matched the publication date restriction: January 2014-June 2022. Papers from March 2020 to June 2022 were included as part of a study update (see the *Literature Update* section). We excluded studies dating from before 2014, as it is argued that around 2015, the DTC-GT market transformed into a second generation in response to a shutdown of the Food and Drug Administration (FDA) of DTC-GT companies [26]. Language requirements were English or Dutch. The included study types were meta-analyses, reviews and systematic reviews, case-control studies, case reports and series, cohort studies, randomized controlled trials, interview and Delphi studies, expert opinion papers, and commentaries. Conference posters and abstracts were excluded.

Studies were included if they described the following: population (P), intervention (I), comparison (C), and outcome (O; PICO; see Table 1) [27]. Citizens (P), excluding animals and microorganisms, who took a DTC-GT for health or lifestyle purposes (I1) or considered taking a DTC-GT for health or lifestyle purposes (I2) were included. In addition, professionals (P2) with expertise in DTC-GTs (I3) were included, among others: health care professionals, ethics experts, and researchers. Studies were restricted to DTC-GTs that purport to improve the consumers’ own health by analyzing their DNA for health and lifestyle purposes. Genealogy and ethnicity testing were excluded. DTC-GTs for prenatal and carrier-screening purposes were also excluded, since the primary aims and subsequent actions of these tests go beyond improving personal health.

Insights were included in this study if they indicated an increased risk or benefit to those citizens who took DTC-GTs when compared with those citizens who did not take a DTC-GT (C1) or those citizens who underwent genetic testing within the conventional health care system. Lastly, the studies must have reported insight into the impact of DTC-GTs along the complete DTC-GT service pathway, including exposure to the offer of DTC-GTs, insight into the validity and utility of DTC-GTs, and the individual and societal impact of a DTC-GT (O). Information about the legal framework of DTC-GTs was not included, since this topic has been reviewed by others and is country specific.

The following steps were taken during the review process: duplicate removal, title and abstract screening using the abovementioned criteria, and full-text screening using the same criteria. The titles and abstracts were screened by 1 researcher (SMO) using the abovementioned criteria. The results were discussed with a second researcher (TLA). Next, 2 researchers (SMO and IKK) divided and screened the remaining full-text papers. When in doubt about inclusion, the researchers discussed this with an additional researcher (MEJ). Those key aspects that determine policy issues were analyzed by 1 researcher (SMO) and later discussed with 3 researchers (MCC, MEJ, and TR). No generative artificial intelligence was used in any portion of the manuscript writing.

**Table 1:** Description of PICO^a^ in this study.

PICO [28]	Included	Excluded
Population	Citizens Experts	Animals Microorganisms
Intervention	Citizens who took a DTC-GT^b^ for health or lifestyle purposes Citizens who considered taking a DTC-GT for health and lifestyle purposes Experts with expertise in DTC-GT services	DTC-GTs for genealogy and ethnicity DTC-GTs for prenatal and carrier screening
Comparison	Citizens who did not take a DTC-GT Citizens who underwent genetic testing within the conventional health care system	N/A^c^
Outcome	The individual and societal impact of DTC-GT services throughout the complete process of the DTC-GT service pathway	Legislation and regulations

^a^PICO: population, intervention, comparison, and outcome.

^b^DTC-GT: direct-to-consumer genetic test.

^c^N/A: not applicable.

### Data Collection and Analysis

The risks and benefits were structured into phases, following the steps that consumers take along the complete DTC-GT service pathway. Studies were analyzed, and the data thus derived were grouped (SMO and IKK; step 2) by means of an iterative process based on the findings of the literature review itself and the model of Mosdøl et al [28]. This model has previously been used to research the effects of direct-to-consumer advertisements, specifically for prescription medicines [29]. The data were divided into the following themes: (1) consumer demographics; (2) test quality, validity, and utility; (3) company features; (4) diseases tested for and health aspects; (5) consumer effects; (6) impact on health care; (7) informational content and advertising; (8) ethics; and (9) general and any remaining information about DTC-GTs.

Information about these themes was gathered to determine those key aspects that lead to policy issues. The key aspects were defined when new themes no longer emerged, that is, when data saturation was achieved. These findings were shared with 5 experts on DTC-GTs, genetic tests, or genomics. Their input was used to further interpret the risks and benefits of DTC-GTs. Next, a framework was composed that structured the key aspects of DTC-GTs that affect potential risks and benefits and engender policy issues (SMO). This was then discussed with 3 researchers (MCC, MEJ, and TR).

### Literature Update

A literature update was performed between March 2020 and June 2022. Novel studies were identified and selected using the same strategy, as described before, differing by only 1 step. During the literature update, we first assessed whether the studies researched themes or topics that were already included in the primary search. If this was the case, they were excluded from the sample. After that, full-text documents were retrieved. The data from the initial literature review (January 2014-March 2020) were supplemented with the potential risks and benefits gleaned from this literature update. Potential risks and benefits that had already been reported in the initial search were viewed as saturated findings, as they did not lead to new policy issues. Those data were therefore not further collected.

### Checklist Development

Ultimately, a checklist was designed to offer guidance in DTC-GT policy issues (SMO and TR) and to evaluate those key aspects that determine product safety, quality, and utility.

Five previously developed and published checklists and frameworks [7,30-33] formed the basis for this checklist. These checklists and frameworks separately do not cover the whole DTC-GT service pathway*.* Therefore, checklist items were selected from these publications to encompass the entire DTC-GT service pathway. The content of all published checklists and frameworks was combined and listed in 1 document. We evaluated each item on this list to see whether it appraised a risk or a benefit identified by our literature review. That way, checklist items were included to match the risks and benefits found in the literature review. Duplicate checklist items were combined into 1 item. When necessary, items were adjusted or supplemented to ensure maximum fit for evaluation of DTC-GT services. The checklist items were further subdivided into themes to improve ease of use.

Questions that evaluate the quality of DTC-GT service elements and informational content on DTC-GT websites were formulated based on the checklist items. For ease of application, the questionnaire was piloted by 3 researchers who analyzed 3 DTC-GT company websites. These 3 companies were selected to reflect the broad variety of services on the market, including 1 international leading DTC-GT company offering a variety of genetic health tests, 1 big international company with a Dutch website offering a variety of genetic health tests, and 1 small and relatively new national Dutch company offering a DTC-GT focused on sport performance and health*.*

## Results

### Integrative Literature Review

**Figure 1:**
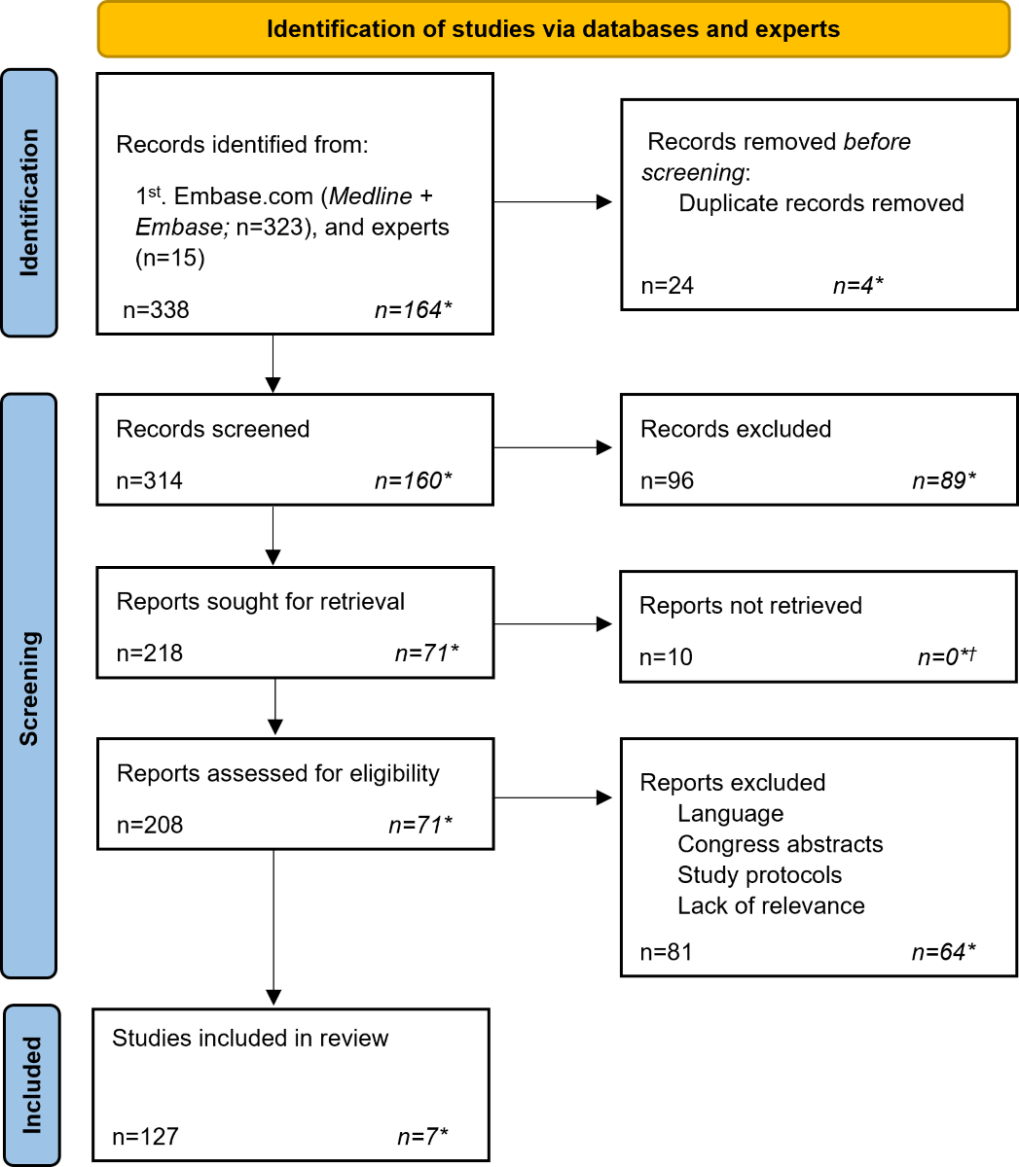
PRISMA diagram describing the reviewing process of the literature search to identify relevant publications between January 2014 and March 2020 and between March 2020 and June 2022. *Papers from the literature update performed between March 2020 and June 2022. †This step was performed after checking whether the studies included in the literature update introduced themes that were not included in the primary search. All 7 papers that yielded novel findings could be retrieved. PRISMA: Preferred Reporting Items for Systematic Reviews and Meta-Analyses.

The literature search resulted in a primary yield of 323 papers (Figure 1). In addition, 15 key papers were received from the 5 experts consulted; however, most of these papers (n=13, 86.7%) were duplicates of the literature search yield. After removal of duplicates (n=24), a total of 314 (97.2%) papers were included for screening. Title and abstract screening and the inability to retrieve full-text papers led to the exclusion of 106 (33.8%) papers. Ultimately, data were collected from 127 (40.4%) papers. The other 81 (25.8%) papers were excluded as they were incorrect document types (Congress posters and abstracts or study protocols), were not published in English or Dutch, or lacked relevance, and this had not been picked up during the first screening. In addition, the literature update yielded 7 papers with novel findings that had not been reported in the initial literature review (see Multimedia Appendix 2 for a list of included papers) [34-40].

### General Findings

DTC-GT services have been researched and discussed in both the descriptive literature, such as expert opinions, and empirical studies, such as questionnaires and interview studies with consumers. The taking of DTC-GTs in case-control and cohort studies was rare. Research mainly focused on various aspects of DTC-GTs, including but not limited to quality and validity, attitudes and perceptions of the public and consumers, the content of the product on offer, experiences of health professionals with DTC-GTs, the utility of the tests, the regulatory landscape surrounding DTC-GTs, and the ethical appraisal of both the advantages and disadvantages of DTC-GTs.

The individual and societal impact of DTC-GT services, caused by their potential risks and benefits, seemed wide ranging. Overall, potential risks were reported more frequently in the literature than potential benefits. The risks and benefits reported included both medical risks, such as inadequately informed health decisions, and nonmedical risks, such as invasion of privacy or obtaining information about as yet unknown family relations. These risks and benefits appeared to be instigated by distinct aspects of DTC-GT services, such as information provision, DNA analysis, and reporting of results.

### The Consumer Journey

**Figure 2:**
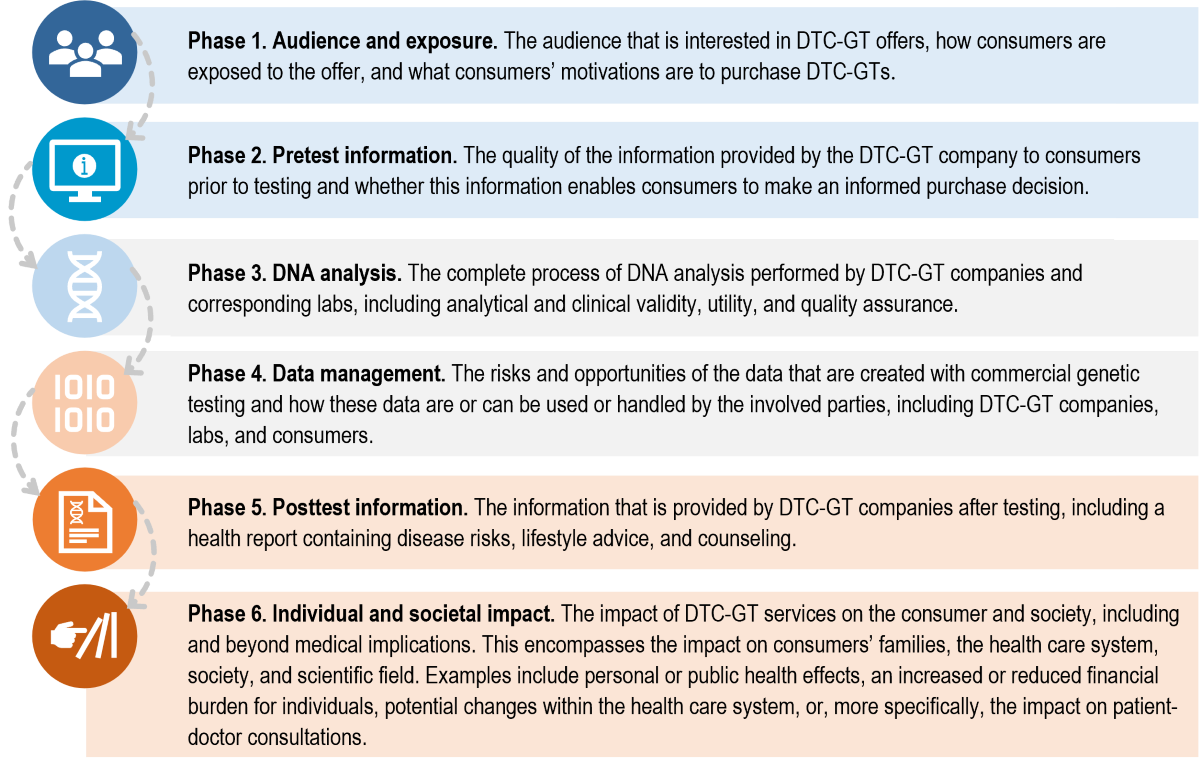
Flow diagram describing the complete process of DTC-GT services, called the consumer journey. As depicted, the consumer journey is broken down into 6 phases. In this study, consumers of the DTC-GT market were both individuals who purchased a DTC-GT or potential consumers who were considering buying the test. DTC-GT: direct-to-consumer genetic test.

Both risks and benefits were structured into phases, following the steps that consumers take when taking DTC-GT services. This led to 6 different phases, collectively called the *consumer journey.* These phases covered the complete process of taking DTC-GT services, from the first exposure to an offer of a DTC-GT service up to the ultimate individual and societal impact of DTC-GT services (depicted in Figure 2). Distinguishing and analyzing the phases of the consumer journey helped obtain insight into the consumer audience, distinct service elements that DTC-GT companies offer their consumers, and the individual and societal impacts of these services*.*

**Figure 3:**
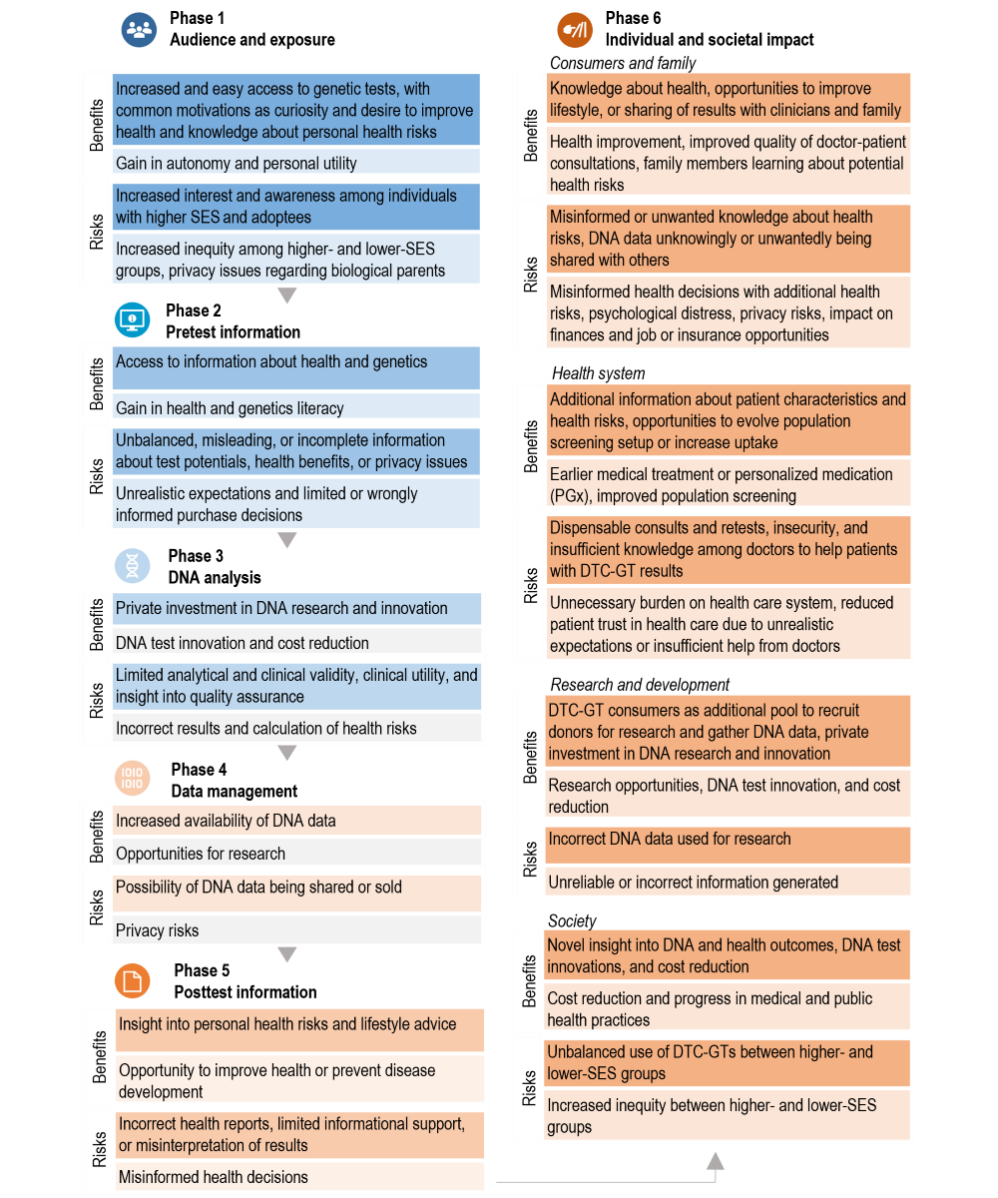
Summary of findings, describing the benefits and risks (darker boxes) of DTC-GTs and their potential impact (lighter boxes) throughout the different phases of the consumer journey. DTC-GT: direct-to-consumer genetic test; PGx: pharmacogenetic tests (DNA tests that predict the patients’ response to specific pharmaceuticals, for example, adverse reactions or insufficient uptake of drugs); SES: socioeconomic status.

Each of these service elements may have distinct risks and benefits (see Figure 3). For example, in phase 1, consumers with common motivations, such as curiosity or the desire to improve health, could benefit from easy access to genetic tests, which may promote autonomy. Interest in DTC-GT services appeared to be more prominent among individuals of a higher socioeconomic status (SES) and also among adoptees; this may negatively impact the health equity and privacy of the biological parents of the latter group.

Phase 2 concerns access to information about health and genetics gathered before purchase of a DTC-GT. This could facilitate health literacy; however, research also points out that information about the potential impact of a DTC-GT, for example, may be unbalanced or misleading. This then impairs the process of informed decision-making among consumers, thereby limiting their autonomy and personal utility.

One benefit of phase 3 DNA analysis may be that private companies are investing in DNA research and innovation, which could boost DNA test innovation and reduce test costs. Yet, aspects such as limited analytical and clinical validity, as well as limited clinical utility and insight into quality assurance, are risks of phase 3 of DTC-GT service pathways. This may cause test results to be erroneous, thus providing consumers with incorrect information about their health risks.

How sensitive genetic data are handled by DTC-GT companies (phase 4), for example, privacy-ensured storage, or potentially shared with third parties can safeguard or impede the privacy of consumers. Genetic data are not unique in the way that they engender privacy issues, yet the data content may be extensive and sometimes concern delicate issues. This makes DNA data sensitive data, which is acknowledged in the General Data Protection Regulation [11].

Although in phase 5, the information provided with the test results may help in making decisions about improving health, thereby improving autonomy, if this information is not clear or the health risks have been calculated based on erroneous results (phase 3), consumers may misinterpret their results and make misinformed health decisions.

Consequently, purchasing and taking DTC-GTs may impact individuals and society (phase 6). Consumers may be empowered to improve their health. However, health risks might arise if sensitive health decisions are based on results of low validity. Moreover, consumers may decide to purchase a test without first fully considering the potential risks and limitations of testing, and upon receiving DTC-GT reports, their expectations may be unmet. Potential risks beyond medical effects include psychological distress as results might be worrying or have an impact on finances and job or insurance opportunities. DTC-GT services may also impair the autonomy of relatives, as DNA is shared within families. The breakthrough in the well-known Golden State Killer cold case, a murder case that was solved by matching the DNA at a crime scene to family members through DTC-GT databanks, is an example of how personal genetic data of relatives, obtained through DTC-GTs, can be used for purposes other than consumers might have opted for [41]. In terms of societal impact, consumers may decide to consult their health care professionals about their results or join large research projects, which may further impact the health care system and research (eg, increased burden, impact on patient trust in health care when medical and DTC-GT results are conflicting, and more DNA data available for research opportunities).

It is important to note that the phases of the consumer journey are often intertwined. For instance, the quality of the information provided after testing (eg, health reports, phase 5) may lead to uncertainties among consumers (individual impact, phase 6), causing them to seek help from professionals in the health care system (societal impact, phase 6). Furthermore, where data storage by DTC-GT companies is either not applicable or guaranteed and executed with adequate safety measurements (phase 4), the privacy of the consumer (individual impact, phase 6) and their family members is preserved (societal impact, phase 6).

### Checklist for Evaluating DTC-GTs

After creating an overview of risks and benefits in each phase of the consumer journey, a checklist for policy guidance based on key aspects of the consumer journey was synthesized. Building on these phases ensured that all steps taken by consumers and all DTC-GT service elements were included in the checklist.

In total, 38 checklist items were derived from published evaluation frameworks and papers [7,30-33]. These items were subdivided into 8 themes (see Table 2):

- Theme I (general DTC-GT service features): 7 checklist items that cover the overall content of a DTC-GT service
- Theme II (DNA analysis and quality assurance): 4 checklist items about the validity and quality assurance of DTC-GT services
- Theme III (privacy and data management): 4 checklist items concerning how consumer privacy and data are managed by DTC-GT companies
- Theme IV (scientific evidence): 3 checklist items relating to the scientific evidence and robustness of DTC-GT services
- Theme V (information about results, interpretation, and consultation): 7 checklist items regarding the information provided about health outcomes assessed, genetics, and results of DTC-GTs
- Theme VI (information about potential consequences of taking a DTC-GT): 6 checklist items that cover information provided about the potential consequences of purchasing DTC-GT services for the consumer, including and beyond medical implications
- Theme VII (presentation of information): 4 checklist items about how information about the previous themes is presented, such as highlighting benefits over risks and vice versa or incorporating opinions of experts or public figures about the usefulness of DTC-GTs
- Theme VIII (informed decision-making): 3 checklist items that help evaluate whether consumers can make informed purchase decisions

Every checklist item reflects a potential risk or benefit found in the integrative literature review. For example, the cost of the test (theme I) applies to the potential risk of increased health disparity between individuals with a higher and a lower SES (consumer journey phase 1). Another example is the DNA test type (theme II), which correlates to the analytical and clinical validity of a DTC-GT and affects the validity of the DTC-GT results (consumer journey phase 3).

Subsequently, 3 researchers tested and administered the checklist in the form of a questionnaire to assess DTC-GT service webshops. This allowed the checklist to be further refined. Questions were formulated for all checklist items. Almost all questions were formulated as closed questions. Potential answers included “yes,” “no,” and “unclear” or “detailed,” “incomplete,” and “missing” when asked to evaluate the information provided about certain key aspects. Open answers were only permitted where the provided closed answer options were deficient (eg, “other, namely…”). Every answer included room for notes and expansion. For details on the layout of the checklist in the form of a questionnaire, see Multimedia Appendix 3.

The 3 researchers reviewed 2 well-known DTC-GT webshops that sell health-related DTC-GT services. On reviewing the webshops, the researchers often came up with different answers when evaluating the same company website. This could imply that the appraisal of webshop content is subjective. Further refinement of the questionnaire may help, although it may not fully compensate for individual differences in how the content of a webshop is perceived.

**Table 2:** Checklist with key items for DTC-GT^a^ service evaluation, grouped by theme.

Theme	Key items
Theme I: general DTC-GT service features (consumer journey phases 1 and 2)	Test costs Assessed health features (eg, disease risks, sports performance, pharmacogenetics) Type of generated report (eg, lifestyle advice, diagnosis) Option to download raw DNA data after testing Option to opt out from receiving certain results Requirement of referral of health professional Residency of the company and the labs
Theme II: DNA analysis and quality assurance (consumer journey phase 3)	DNA test type (eg, sequencing, single-nucleotide polymorphism [SNP] array) Quality assurance in labs that perform DTC-GTs (eg, International Standardization Organization (ISO)/Clinical Laboratory Improvement Amendments (CLIA) certification) Information about how much and which genes or DNA variants will be analyzed All relevant background information of consumers considered
Theme III: privacy and data management (consumer journey phase 4)	Presence of a privacy policy on the website Management of consumers’ personal and genetic data by the company (eg, duration of storage, will the data be shared with or sold to third parties) Management of consumers’ samples after DNA analysis by the company (eg, duration of storage, what will happen to the samples upon bankruptcy) Option to withhold storage, sharing, and selling of samples and data
Theme IV: scientific evidence and robustness (consumer journey phase 3)	Methods based on reliable scientific evidence (DNA test type) Robustness of scientific evidence underlying the chosen methods (eg, novel gene-disease interactions vs well-studied gene-disease interactions) Collaboration with scientific partners (eg, academic research groups, prominent researchers from universities)
Theme V: information about results, interpretation, and consultation (consumer journey phase 5)	Information about the assessed health feature (eg, disease burden, prevalence) Information about how to interpret results Information about the robustness of results Possible actions in the case of a positive finding (eg, treatment options) Option to consult a genetic professional at the company Recommendation to consult a health care professional to discuss test results Referral to companies or websites for additional analysis on raw DNA data (ie, third-party analyzers)
Theme VI: information about potential consequences of taking a DTC-GT (consumer journey phases 3, 5, and 6)	DTC-GT results informing and impacting future health decisions and health behavior DTC-GT results impacting insurance policies (eg, health insurance) DTC-GT results impacting family (eg, revelation of family health risks or unknown family relations) DTC-GT having other consequences beyond medical purposes (eg, feeling of anxiety or relief following test results) DTC-GT results altering due to future technological innovations (eg, new health results) DTC-GT service company investing in or performing own research
Theme VII: presentation of information (consumer journey phases 3 and 5)	Usage of fear Usage of public figures or medical experts to convince consumers into buying the test Balanced information about advantages, usefulness, disadvantages, and risks of taking a DTC-GT Clear distinction between health outcomes: (1) for which (proven effective) treatments or interventions are available, (2) whose outcome can be partially improved, (3) for which treatment is not yet available
Theme VIII: informed decision-making (consumer journey phases 3 and 5)	Information that analyzing the DNA of others without consent is ethically irresponsible or punishable or both Active confirmation of informed decision (eg, via a pop-up) Generally clear, understandable, complete, and easy to retrieve information provided to make an informed decision (eg, privacy policy)

^a^DTC-GT: direct-to-consumer genetic test.

## Discussion

### Principal Findings

Following the results of an integrative literature review, this paper described the potential risks and benefits of DTC-GT services that may cause policy issues. The risks and benefits are wide ranging. They include medical risks and benefits, such as inadequately informed health decisions and improved knowledge about genetics and health, as well as nonmedical risks and benefits, such as invasion of privacy or obtaining information about as yet unknown family relations. These risks and benefits seem to be linked to distinct aspects of DTC-GT services, such as information provision, DNA analysis, and reporting of results. By structuring the risks and benefits into phases, following the consumers’ steps when taking a DTC-GT service, 6 phases can be distinguished, which are collectively called the consumer journey*.* The consumer journey provides insight into the consumer audience, distinct service elements that DTC-GT companies provide for their consumers, and the individual and societal impacts of these services. Distinguishing different steps in the DTC-GT service process reveals multiple aspects of the offers of DTC-GT services that engender policy issues and may require policy guidance in the future. The checklist that was introduced in this study provides a backbone for policy guidance for DTC-GT services. To ensure coverage across all DTC-GT service elements, checklist items were included to evaluate all phases of the consumer journey. Each checklist item covers a risk or a benefit found in the literature review. By reviewing and extensively discussing the medical, ethical, social, and technological issues of DTC-GTs, the checklist translates current scientific knowledge into a concise and helpful instrument for policy makers, regulators, and law enforcers.

### Implications

In its current form, the checklist summarizes crucial items that may affect individuals or society and that can trigger policy issues and may call for intervention. Combined with insight into the phases of the consumer journey, this could help different stakeholders comprehend the potential risks and benefits of the DTC-GT service market. Horton et al [42] structured the process of DTC-GT services similarly to the consumer journey. In their model, the risks in the DTC-GT process were described by presenting fictitious cases and what to discuss with patients in a clinical setting. Yet, their study focused mainly on the impact of technical and quality limitations after a consumer chooses to purchase a test, while we included the impact of all aspects of DTC-GT services, for example, information provision and informed purchase decisions, data management and privacy, and the impact on family members. The current combination of the checklist and the consumer journey could be helpful for a broader audience, in particular regulators, law enforcers, and policy makers within professional or patient organizations who are expected to safeguard their citizens or patients. To ensure a clear strategy on how to act in the DTC-GT market, it may be fruitful to design a process that incorporates the checklist and helps assign specific responsibility holders to specific policy issues. Policy makers at the national level may facilitate this by delegating tasks. If there is no national party to take on this directorial role, regulators, law enforcers, and policy makers may use the checklist to set their own agenda, preferably in collaboration with relevant stakeholders.

By using the checklist, policy makers can structurally review the DTC-GT services on offer to the residents of their country. This will help them determine societal and public health hazards and reveal aspects of DTC-GT services or highlight specific providers that call for intervention. Furthermore, it could aid in monitoring potentially useful genetic testing applications for uptake in health care and thereby strive to reduce the risk of inequity. Considering the roles and responsibilities of policy makers, this checklist may prove especially useful in monitoring the risks of the current market and protecting citizens from harm. For instance, public health agencies, patient and consumer organizations, and professional associations may choose to take responsibility for monitoring the tests on offer, as well as consumer issues, such as uninformed purchase decisions leading to unexpected results, or results of limited validity.

Furthermore, policy experts may review the legislation within a country with the checklist items to check for leads for law enforcement or gaps in regulations that call for novel policy. This is expected to be country specific, as the diverse service elements provided by DTC-GT companies, such as data protection and in vitro diagnostic medical devices, fall within distinct regulatory areas in Europe and the United States [11,22-24,43].

Several steps can still be taken to refine the checklist. Although all items on the checklist can lead to potential risks for individuals and society, the size of the risk or benefit and its subsequent impact may differ. Consequently, the next step in the development and application of this checklist is to prioritize checklist items. In doing this, it is important to collect and compare empirical evidence on the risks of DTC-GT services. Important factors that may influence prioritizing the checklist items include:

- The probability that a risk will occur—rare versus often
- The impact of the risk—wrongly informed health decisions versus learning about as yet unknown family members
- The availability of clear regulations that enable law enforcement—available versus absent

Additionally, the checklist could be used to determine crucial aspects of an informed purchase decision. To achieve this, there should be more focus on improving the current version of the checklist to facilitate ease of use for consumers. This could include items such as a visual online tool or decision aid and the use of accessible language. Information regarding the potential impact of gaining health insights through DTC-GT services may help empower consumers decide whether they, indeed, want to purchase and use a DTC-GT service, from which provider, and which health outcomes they do or do not want to learn more about. It may also help them understand how to interpret results, the impact of the results and consequent health decisions, and how their data are handled. This is essential, as the information on DTC-GT service websites is often misleading or incomplete [44,45].

### Future Directions

Deciding on how to act in the online, complex, and diverse DTC-GT service market and to strike a balance between ensuring public safety and maintaining autonomy is challenging. To move forward, it is key that stakeholders maintain an ongoing discussion about the potential impact of these products on the public and about what impact should be desired or should be avoided. Stakeholders, such as policy makers, law enforcers, regulators, health care professionals, and members of the public, should be informed by ongoing research.

DTC-GT services combine elements of many mHealth products [46]. Specific elements of DTC-GT services can be seen in many mHealth technologies, such as easily retrievable health information, insight into personal health and disease risk, and products that enable preventive actions and self-management and empower citizens. Due to the sensitive nature of genetic data, DTC-GT services present additional challenges. These include issues regarding the reidentification and sharing of DNA within families, leading to privacy issues and concerns regarding the “right not to know” among consumers and their family members, and the challenges of comprehending and acting on complex personal risk information.

DTC-GT services and mHealth products in general challenge traditional distinctions between clinical care and self-promoted well-being: what is a medical matter, and what are personal lifestyle choices? These services raise questions about reimbursement policies, for instance, insurance companies that use devices to monitor customers’ lifestyle and ultimately to adapt their premium. Another challenge lies in defining which devices require special certification because of specific safety issues [47]. Unraveling existing international policies on diverse mHealth products and how these policies affect the uptake and impact of mHealth products may aid policy makers in deciding how to respond to the market. Furthermore, comparing technologies and their potential consequences helps determine whether and how the public might be empowered or impeded.

The internet enables easy communication and the dissemination of information, which is a strong suit of DTC-GT service companies. To empower citizens and ensure informed purchase decisions, it is key that consumers be well informed about the potential impact of service aspects in all phases of the consumer journey. Aspects such as privacy and data management, scientific evidence, and information about how to use DTC-GTs and interpret their results are relevant to ensuring safety and maintaining autonomy. Studies that show variance in result comprehension [48], emphasis on positive aspects and negligence of potential risks by companies [45], limited consideration of potential risks by consumers prior to testing [49], and unmet expectations of DTC-GT service potential [49] suggest that the quality of the information currently available on DTC-GTs should be improved [44]. Further research is required to understand the communication strategies of companies, to point out information needs, and to determine how these needs should be met in order to support consumers in their decisions about DTC-GT services. The framework presented here could aid in distinguishing the various aspects of a service about which information can be provided, which information empowers consumers in their purchase decisions, and which information can be easily misunderstood.

In understanding the need for information provision, an important question is who should provide this support. This could include general practitioners [50], clinical geneticists and genetic counselors [51], nurses [52], pharmacists [53], government and public health institutes, and DTC-GT service providers. Several factors may impact the choice of which strategy would be fitting for dissemination. Examples of these factors include the media via which consumers are often exposed to offers of tests, such as TikTok [34] and YouTube [54], and consumer motivations to purchase, including the motivation to improve their own health [55] or an adoptee’s health without knowledge of the family disease history [56]. Additional research should help clarify how consumers can be empowered in their decisions prior to and after purchasing a DTC-GT service [46].

### Limitations

This study outlined the potential risks and opportunities in the consumer journey of DTC-GT services based on an integrative literature review. However, we were unable to determine the extent of either the risks or the opportunities or to point out which risks and opportunities should have priority over others. Furthermore, due to the complexities involved, the study lacks in-depth insight into which legislation applies to the diverse aspects of the services in each specific country. Answers to these questions will provide insight into policy decisions related to the DTC-GT service market. To gain knowledge of this, more empirical evidence is needed on items such as the validity of DTC-GTs, the wanted or unwanted effects of taking DTC-GTs, or factors that codetermine whether risks and opportunities will occur.

Second, the synthesized checklist is not a tool that policy makers, law enforcers, and regulators can directly apply to any policy. Therefore, the checklist is not yet a ready-made instrument. However, in their current forms, the checklist and consumer journey may together serve as a framework for multiple policy needs. The checklist has room for further refinement, depending on policy needs, such as insight into information provision on DTC-GT websites or appraisal of legislation governing distinct aspects of the service. Potentially, future checklists could be designed to focus specifically on 1 jurisdiction or 1 aspect of the consumer journey governed by specific legislation (eg, clinical validity under In Vitro Diagnostics Regulation [11]).

Third, the findings of our literature search may be biased toward the risks of the DTC-GT market. This may be reflected in the summary of findings and the checklist. The publications included indicate a greater focus on the risks of the DTC-GT service market than on the opportunities. This could be partially due to the fact that experts are rather skeptical about the potential health benefits of the DTC-GT market because of the immaturity of scientific evidence on the validity and utility of the tests offered by companies, for instance.

A shift in the study approach toward the effects of DNA testing for disease prevention, personalized medicine, or increased public education could reveal more potential opportunities for the DTC-GT market. For example, research on pharmacogenetic testing and health outcomes may provide further insight into the potential benefits of the commercial genetic testing market [57]. Moreover, the findings of these studies could also enhance knowledge of those elements that are key to improving the effectiveness and safety of commercially available genetic testing services.

Fourth, this study excluded DTC-GTs for preconception carrier testing and prenatal testing. However, these tests could impact public health indirectly, as they could affect reproductive decisions. Couples may decide to not have children or seek additional medical care in conceiving a child. In addition, other issues may result from carrier or prenatal genetic testing, such as informed reproductive decisions, sex selection, and stigmatization or discrimination against disabled individuals [58,59]. Insight into issues of DTC-GT services for reproductive purposes may be relevant for policy experts. Many issues found in this study may be applicable for carrier or prenatal DTC-GTs, such as questions regarding privacy, validity, and learning about unexpected disease risks. Presumably, the need for informational support may be of comparable importance, or even greater importance, to consumers who are considering taking carrier or prenatal DTC-GTs and then using the results in their reproductive decisions.

### Conclusion

This study summarized the risks and benefits associated with distinct DTC-GT aspects at a personal and a public level. Both the consumer journey and the checklist break the offer of a DTC-GT service down into key aspects that may impact and compromise the safety and autonomy of the individual and of public health. This framework helps policy makers, regulators, and law enforcers develop methods to interpret, assess, and act in the DTC-GT service market. This could serve as a backbone for future research into key themes such as the need for provision of information. The checklist could be further developed into a decision support tool to empower consumers in their decisions both before and after purchasing a DTC-GT service.

## Appendix

Multimedia Appendix 1 Search strategy for DTC-GTs in Embase.com (Medline + Embase). DTC-GT: direct-to-consumer genetic test.

Multimedia Appendix 2 List of all the articles included in the literature study. The list combines the articles from both the initial literature review and the update.

Multimedia Appendix 3 DTC-GT webshop questionnaire. DTC-GT: direct-to-consumer genetic test.

## Abbreviations

DTC-GT: direct-to-consumer genetic test
mHealth: mobile health
PICO: population, intervention, comparison, and outcome
SES: socioeconomic status

## Data Availability

The data sets generated and analyzed during this study are available from the corresponding author upon reasonable request. A list of all the included papers can be found in Multimedia Appendix 2.
