# Supplementary material for: Policy Guidance for Direct-to-Consumer Genetic Testing Services: Framework Development Study
Source: J Med Internet Res. 2024 Jul 17;26:e47389. doi: 10.2196/47389 (PMC11292153; doi:10.2196/47389)
Supplement: Multimedia Appendix 1 [file jmir_v26i1e47389_app1.docx]

| **Steps** | **Search string** |
| --- | --- |
| **Ultimate search:** (#34 OR #35 OR #36 OR #39 OR #42) AND [2014-2020]/pv | |
| #1 | ‘direct to consumer;:ti AND ‘genetic screening’/exp OR ‘genetic test’: ti,ab OR ‘genetic health test’:ti,ab OR ‘dna-test’:ti,ab OR ‘genetic screening’:ti,ab  OR 'direct to consumer*':ti,ab AND 'genetic screening'/exp/mj OR 'genetic test*':ti OR 'genetic health test*':ti OR 'dna-test*':ti OR 'genetic screening*':ti  OR ‘direct to consumer genetic testing’/exp |
|  | |
| #2 | 'clinical study'/exp OR 'intervent*':ti |
| #3 | 'evidence based practice'/exp OR 'eviden*':ti |
| #4 | 'accuracy'/exp OR 'accura*':ti |
| #5 | 'diagnostic accuracy'/exp |
| #6 | 'reliability'/exp OR 'reliab*':ti |
| #7 | 'controlled study'/exp OR 'controll*':ti |
| #8 | 'prediction and forecasting'/exp OR 'prognostic value*':ti OR 'prognosis':ti OR ('predict*':ti AND 'forecast*':ti) |
| #9 | 'harm reduction'/exp OR harm:ti,ab OR 'harmfull*':ti,ab |
| #10 | 'weakness'/exp OR 'weakness*':ti,ab |
| #11 | 'disadvantage*':ti,ab OR 'advantage*':ti,ab |
| #12 | 'legislation and jurisprudence'/exp OR 'legislation*':ti,ab |
| #13 | 'opportunit*':ti,ab AND 'challenge*':ti,ab |
| #14 | 'pitfall*':ti,ab |
| #15 | 'third party':ti |
| #16 | 'impact*':ti |
| #17 | 'genetic counseling'/exp/mj OR 'utilizat*':ti OR 'utility':ti |
| #18 | 'prevention'/exp OR 'prevent*':ti |
| #19 | 'added*':ti,ab |
| #20 | 'sport'/exp OR sport:ti OR 'athlet*':ti |
| #21 | 'diseases'/exp/mj |
| #22 | 'nutrition'/exp OR 'diet*':ti OR 'nutrition*':ti |
| #23 | 'diet therapy'/exp |
| #24 | 'quality control'/exp OR 'quality*':ti |
| #25 | 'health care quality'/exp/mj |
| #26 | 'practice guideline'/exp/mj OR 'guideline*':ti |
| #27 | 'law'/exp OR law:ti OR laws:ti OR 'legislat*':ti OR 'privacy':ti OR 'legal*':ti OR 'regulat*':ti |
| #28 | 'risk assessment'/exp OR 'risk*':ti |
| #29 | ‘elsi:ti OR ('ethical*':ti AND 'legal*':ti AND 'social*':ti AND 'issue*':ti) |
| #30 | 'lifestyle'/exp OR 'lifestyle*':ti |
| #31 | 'marketing'/exp OR 'marketing*':ti |
| #32 | 'validity'/exp OR 'valid*':ti OR 'comparison*':ti |
| #33 | 'threat'/exp/mj OR 'threat*':ti OR 'benefit*':ti OR 'opportune*':ti OR 'quality*':ti |
|  | |
| #34 | #1 AND (#2 OR #3 OR #4 OR #5 OR #6 OR #7 OR #8 OR #9 OR #10 OR #11 OR #12 OR #13 OR #14 OR #15 OR #16 OR #17 OR #18 OR #19 OR #20 OR #21 OR #22 OR #23 OR #24 OR #25 OR #26 OR #27 OR #28 OR #29 OR #30 OR #31 OR #32 OR #33 OR ‘review’/it) |
|  | |
| #35 | 'direct to consumer*':ti AND (‘pharmacogenetic testing’/exp/mj OR ‘guideline*’:ti) |
|  | |
| #36 | 'screening test'/exp/mj AND ('genetic screening'/exp/mj OR 'genetic test*':ti OR 'genetic health test*':ti OR 'dna-test*':ti OR 'genetic screening*':ti) |
|  | |
| #37 | (elsi:ti OR ('ethical*':ti AND 'legal*':ti AND 'social*':ti AND 'issue*':ti)) OR ('direct-to-consumer advertizing'/exp) OR ('direct to consumer*':ti,ab) |
| #38 | ('predictive value'/exp OR 'predictive value*':ti,ab) OR ('behavior change'/exp OR 'behavior change*':ti,ab) OR ('lifestyle modification'/exp OR 'lifestyle modification*':ti,ab) OR ('health benefit*':ti,ab) |
| #39 | #37 AND #38 |
|  | |
| #40 | ('genetic screening'/exp/mj OR 'genetic test*':ti OR 'genetic health test*':ti OR 'dna-test*':ti OR 'genetic screening*':ti) OR ('pharmacogenetic testing'/exp OR 'pharmacogenet*':ti) OR ('screening test'/exp/mj) |
| #41 | (elsi:ti OR ('ethical*':ti AND 'legal*':ti AND 'social*':ti AND 'issue*':ti)) OR ('predictive value'/exp/mj OR 'predictive value*':ti) OR ('behavior change'/exp/mj OR 'behavior change*':ti) OR ('lifestyle modification'/exp/mj OR 'lifestyle modification*':ti) OR ('health benefit*':ti) |
| #42 | #40 AND #41 |

**Multimedia Appendix 1:** search strategy direct-to-consumer genetic testing in Embase.com (Medline + Embase)
