# Supplementary material for: Policy Guidance for Direct-to-Consumer Genetic Testing Services: Framework Development Study
Source: J Med Internet Res. 2024 Jul 17;26:e47389. doi: 10.2196/47389 (PMC11292153; doi:10.2196/47389)
Supplement: Multimedia Appendix 2 [file jmir_v26i1e47389_app2.docx]

**Multimedia Appendix 2**. List of all the articles included in the integrative literature review. The list combines the articles from both the initial literature review and the update.

| **Nr.** | **Authors** | **Year** | **Title** | **Journal** |
| --- | --- | --- | --- | --- |
| 1 | ACOG | 2017 | Committee Opinion No. 724: Consumer Testing for Disease Risk | Obstet and Gynec |
| 2 | AMCG Board or Directors | 2016 | Direct-to-consumer genetic testing: a revised position statement of the American College of Medical Genetics and Genomics | Genet Med |
| 3 | Agurs-Collins, T., et al. | 2015 | Public Awareness of Direct-to-Consumer Genetic Tests: Findings from the 2013 U.S. Health Information National Trends Survey | J Cancer Educ. |
| 4 | Allyse, M. A., et al. | 2018 | Direct-to-Consumer Testing 2.0: Emerging Models of Direct-to-Consumer Genetic Testing | Mayo Clin Proc. |
| 5 | Apathy, N. C., et al. | 2018 | Trends and Gaps in Awareness of Direct-to-Consumer Genetic Tests From 2007 to 2014 | Am J Prev Med. |
| 6 | Artin, M. G., et al. | 2019 | Cases in precision medicine: When patients present with direct-to-consumer genetic test results | Ann Intern Med. |
| 7 | Austin, J. | 2015 | The effect of genetic test-based risk information on behavioral outcomes: A critical examination of failed trials and a call to action | Am J Med Genet A. |
| 8 | Badalato, L., et al. | 2017 | Third party interpretation of raw genetic data: An ethical exploration | Eur J Hum Genet. |
| 9 | Baptista, N. M., et al. | 2016 | Adopting genetics: Motivations and outcomes of personal genomic testing in adult adoptees | Genet Med. |
| 10 | Barton, M. K. | 2017 | Health behaviors not significantly changed by direct-to-consumer genetic testing | CA Cancer J Clin. |
| 11 | Basch, C.H. et al. ^a^ | 2021 | A content analysis of direct-to-consumer DNA testing on TikTok | J Community Genet |
| 12 | Bates, M. | 2018 | Direct-to-consumer genetic testing: Is the public ready for simple, at-home DNA tests to detect disease risk? | IEEE Pulse. |
| 13 | Bertolotti, M. | 2015 | Opportunities, risks, and limitations of genetic testing: Looking to the future from patients' point of view | Mayo Clin Proc |
| 14 | Bird, S. | 2014 | Genetic testing: medico-legal issues | Forensic Sci Int |
| 15 | Bloss, C. S., et al. | 2014 | Direct-to-consumer pharmacogenomic testing is associated with increased physician utilisation | J Med Genet |
| 16 | Boeldt, D. L., et al. | 2015 | Influence of individual differences in disease perception on consumer response to direct-to-consumer genomic testing | Clin Genet |
| 17 | Broady, K. M., et al. | 2018 | Predictors of adverse psychological experiences surrounding genome-wide profiling for disease risk | J Community Genet |
| 18 | Brunstein, J. | 2016 | Perils, pitfalls, and promise of direct-to-consumer genetic testing | MLO Med Lab Obs |
| 19 | Bruke, S. et al. ^a^ | 2021 | Genetic counselor approaches to BRCA1/2 direct-to-consumer genetic testing results | J Genet Couns |
| 20 | Burke, W. and Trinidad, S. B. | 2016 | The deceptive appeal of direct-to-consumer genetics | Ann Intern Med |
| 21 | Camp, K. M. and Trujillo, E. | 2014 | Position of the academy of nutrition and dietetics: Nutritional genomics | J Acad Nutr Diet |
| 22 | Camporesi, S. and McNamee, M. J. | 2016 | Ethics, genetic testing, and athletic talent: Children’s best interests, and the right to an open (athletic) future | Physiol Genomics |
| 23 | Carere, D. A., et al. | 2017 | Prescription medication changes following direct-to-consumer personal genomic testing: Findings from the Impact of Personal Genomics (PGen) Study | Genet Med |
| 24 | Carere, D. A., et al. | 2015 | The impact of direct-to-consumer personal genomic testing on perceived risk of breast, prostate, colorectal, and lung cancer: Findings from the PGen study | BMC Med Genomics |
| 25 | Carroll, N. M., et al. | 2019 | Demographic differences in the utilization of clinical and direct-to-consumer genetic testing | J Genet Couns |
| 26 | Charbonneau, J., et al. | 2019 | Public reactions to direct-to-consumer genetic health tests: A comparison across the US, UK, Japan and Australia | Eur J Hum Genet |
| 27 | Christofides, E. and O’Doherty, K. | 2016 | Company disclosure and consumer perceptions of the privacy implications of direct-to-consumer genetic testing | New Genetics and Society |
| 28 | Clayton, E. W. | 2020 | Be Ready to Talk with Parents about Direct-to-Consumer Genetic Testing | JAMA Pediatr |
| 29 | Cornel, M. C., et al. | 2014 | The challenge of implementing genetic tests with clinical utility while avoiding unsound applications | J Community Genet |
| 30 | Covolo, L., et al. | 2015 | Internet-Based Direct-to-Consumer Genetic Testing: A Systematic Review | J Med Internet Res |
| 31 | Dasgupta, S. | 2017 | Medical Genetics Ethics Case Collection: Discussion Materials for Medical Students in the Genomic Era | MedEdPORTAL |
| 32 | De, S., et al. | 2019 | Information Provided to Consumers about Direct-to-Consumer Nutrigenetic Testing | Public Health Genomics |
| 33 | Delaney, S. K. and Christman, M. F. | 2016 | Direct-to-Consumer Genetic Testing: Perspectives on Its Value in Healthcare | Clin Pharmacol Ther |
| 34 | Dinulos, M. B. P. and S. E. Vallee | 2020 | The Impact of Direct-to-Consumer Genetic Testing on Patient and Provider | Clin Lab Med |
| 35 | Etchegary, H. | 2014 | Public attitudes toward genetic risk testing and its role in healthcare | Per Med |
| 36 | Farmer, M. B., et al. | 2019 | Errors in Genetic Testing: The Fourth Case Series | Cancer J |
| 37 | Floris, M., et al. | 2020 | Direct-to-consumer nutrigenetics testing: An overview | Nutrients |
| 38 | Franceschini, N., et al. | 2018 | Genetic Testing in Clinical Settings | Am J Kidney Dis |
| 39 | Friez, M. J. | 2018 | Attention: Direct-To-Consumer patrons: Proceed with caution | Genet Med |
| 40 | Garrison, N. A. and Non, A. L. | 2014 | Direct-to-consumer genomics companies should provide guidance to their customers on (not) sharing personal genomic information | Am J Bioeth |
| 41 | Gollust, S. E., et al. | 2017 | Consumer Perspectives on Access to Direct-to-Consumer Genetic Testing: Role of Demographic Factors and the Testing Experience | Milbank Q |
| 42 | Guasch-Ferré, M., et al. | 2018 | Nutritional Genomics and Direct-to-Consumer Genetic Testing: An Overview | Adv Nutr |
| 43 | Haga, S. B., et al. | 2019 | Primary care physicians’ knowledge, attitudes, and experience with personal genetic testing | J Pers Med |
| 44 | Hall, J. A., et al. | 2017 | Transparency of genetic testing services for health, wellness and lifestyle': Analysis of online prepurchase information for UK consumers | Eur J Hum Genet |
| 45 | Harris, A., et al. | 2014 | Autobiologies on YouTube: Narratives of direct-to-consumer genetic testing | New Genet Soc |
| 46 | Hayashi, M., et al. | 2018 | Effectiveness of personal genomic testing for disease-prevention behavior when combined with careful consultation with a physician: a preliminary study | BMC Res Notes |
| 47 | Hazel, J. W. and Slobogin, C. | 2018 | Who Knows What, and When?: A Survey of the Privacy Policies Proffered by U.S. Direct-to-Consumer Genetic Testing Companies | Cornell J Law Public Policy |
| 48 | Hendricks-Sturrup, R. M. and Lu, C. Y. | 2019 | Direct-to-consumer genetic testing data privacy: Key concerns and recommendations based on consumer perspectives | J Pers Med |
| 49 | Hendricks-Sturrup, R. M., et al. | 2019 | Direct-to-Consumer Genetic Testing and Potential Loopholes in Protecting Consumer Privacy and Nondiscrimination | JAMA |
| 50 | Hogarth, S. and Saukko, P. | 2017 | A market in the making: the past, present and future of direct-to-consumer genomics | New Genetics and Society |
| 51 | Horne, J., et al. | 2018 | A Systematic Review of Genetic Testing and Lifestyle Behaviour Change: Are We Using High-Quality Genetic Interventions and Considering Behaviour Change Theory? | Lifestyle Genom |
| 52 | Horton, R., et al. | 2019 | Direct-to-consumer genetic testing | BMJ |
| 53 | Huml, A. M., et al. | 2020 | Consistency of Direct-to-Consumer Genetic Testing Results Among Identical Twins | Am J Med |
| 54 | Ibrahim, R., et al. | 2016 | Omics for personalized medicine: defining the current we swim in | Expert Rev Mol Diagn |
| 55 | Jackson, L., et al. | 2014 | Guidance for patients considering direct-to-consumer genetic testing and health professionals involved in their care: Development of a practical decision tool | Fam Pract |
| 56 | Jonas, M. C., et al. | 2019 | Physician experience with direct-to-consumer genetic testing in Kaiser permanente | J Pers Med |
| 57 | Kalokairinou, L., et al. | 2019 | Attitudes and experiences of European clinical geneticists towards direct-to-consumer genetic testing: a qualitative interview study | New Genetics and Society |
| 58 | Kalokairinou, L., et al. | 2020 | It's much more grey than black and white': clinical geneticists' views on the oversight of consumer genomics in Europe. | Per Med |
| 59 | Kiselev, Y., et al. | 2019 | Opportunities and challenges with direct-to-consumer genetic tests in personalised care | Int J Clin Pharmacology |
| 60 | Klein, E. L. Z. | 2014 | The Increasing Role of Genetics and Genomics in Women's Health | Nurs Womens Health |
| 61 | Koeller, D. R., et al. | 2017 | Utilization of Genetic Counseling after Direct-to-Consumer Genetic Testing: Findings from the Impact of Personal Genomics (PGen) Study | J Genet Couns |
| 62 | Laestadius, L. I., et al. | 2017 | All your data (effectively) belong to us: Data practices among direct-to-consumer genetic testing firms | Genet Med |
| 63 | Lee, H. et al. ^a^ | 2021 | Adult adoptees and their use of direct-to-consumer genetic testing: Searching for family, searching for health | J Genet Couns |
| 64 | Levenson, D. | 2017 | Few direct-to-consumer test users receive genetic counseling: More consumers discuss results with primary care physicians | Am J Med Genet A |
| 65 | Loi, M. | 2016 | Direct to consumer genetic testing and the libertarian right to test | J Med Ethics |
| 66 | Lu, M., et al. | 2017 | Pharmacogenetic testing through the direct-to-consumer genetic testing company 23andMe | BMC Med Genomics |
| 67 | Mahon, S. M. | 2018 | Direct-to-Consumer Genetic Testing: Helping Patients Make Informed Choices | Clin J Oncol Nurs |
| 68 | McGowan, M. L., et al. | 2014 | Gatekeepers or intermediaries? The role of clinicians in commercial genomic testing | PLoS One |
| 69 | McGrath, S. P., et al. | 2016 | Comprehension and data-sharing behavior of direct-to-consumer genetic test customers | Public Health Genomics |
| 70 | McGrath, S. P., et al. | 2019 | Are providers prepared for genomic medicine: interpretation of Direct-to-Consumer genetic testing (DTC-GT) results and genetic self-efficacy by medical professionals | BMC Health Serv Res |
| 71 | Mena, C. and Terry, S. F. | 2017 | A New Day Dawns for Direct-to-Consumer Marketing | Genet Test Mol Biomarkers |
| 72 | Metcalfe, S. A., et al. | 2018 | Australians’ views on personal genomic testing: focus group findings from the Genioz study | Eur J Hum Genet |
| 73 | Middleton, A., et al. | 2017 | Direct-to-consumer genetic testing: Where and how does genetic counseling fit? | Per Med |
| 74 | Moscarello, T., et al. | 2019 | Direct-to-consumer raw genetic data and third-party interpretation services: more burden than bargain? | Genet Med |
| 75 | Narod, S.A. et al. ^a^ | 2021 | The screen project: Guided direct-to-consumer genetic testing for breast cancer susceptibility in Canada | Cancers |
| 76 | Nelson, B. | 2016 | The big sell: Direct-to-consumer tests promise patients more abundant and accessible information, but potential pitfalls abound | Cancer Cytopathol |
| 77 | Nelson, B. | 2016 | Greater goods?: Direct-to-consumer testing companies are making a broader case for societal benefits, but not everyone is sold | Cancer Cytopathol |
| 78 | Nelson, S. C. and Fullerton, S. M. | 2018 | Bridge to the Literature? Third-Party Genetic Interpretation Tools and the Views of Tool Developers | J Genet Couns |
| 79 | Nhan, H. et al. ^a^ | 2020 | Allele frequencies of two MUTYH variants, Y179C, and G396D, in a direct-to-consumer genetic database | J Clin Oncology |
| 80 | Nielsen, D. E., et al. | 2017 | Diet and exercise changes following direct-to-consumer personal genomic testing | BMC Med Genomics |
| 81 | Nielsen, D. E., et al. | 2014 | Perceptions of genetic testing for personalized nutrition: A randomized trial of DNA-based dietary advice | J Nutrigenet Nutrigenomics |
| 82 | Niemiec, E., et al. | 2017 | Current ethical and legal issues in health-related direct-to-consumer genetic testing | Per Med |
| 83 | Nordgren, A. | 2014 | Neither as harmful as feared by critics nor as empowering as promised by providers: Risk information offered direct to consumer by personal genomics companies | J Community |
| 84 | Olfson, E., et al. | 2016 | Implications of personal genomic testing for health behaviors: The case of smoking | Nicotine Tob Res |
| 85 | Oliveri, S., et al. | 2016 | Anxiety delivered direct-to-consumer: Are we asking the right questions about the impacts of DTC genetic testing? | J Med Genet |
| 86 | Oliveri, S., et al. | 2015 | Living at Risk: Factors That Affect the Experience of Direct-to-Consumer Genetic Testing | Mayo Clin Proc |
| 87 | Ostergren, J. E., et al. | 2015 | How Well Do Customers of Direct-to-Consumer Personal Genomic Testing Services Comprehend Genetic Test Results? Findings from the Impact of Personal Genomics Study for the PGen Study Group | Public Health Genomics |
| 88 | Park, J. Y., et al. | 2019 | Privacy in Direct-to-Consumer Genetic Testing | Clin Chem |
| 89 | Paul, N. W., et al. | 2014 | Captious certainties: Makings, meanings and misreadings of consumer-oriented genetic testing | J Community Genet |
| 90 | Petersen, L. M. and J. A. Lefferts | 2020 | Lessons Learned from Direct-to-Consumer Genetic Testing | Clin Lab Med |
| 91 | Phillips, A. M. | 2017 | Reading the fine print when buying your genetic self online: direct-to-consumer genetic testing terms and conditions | New Genetics and Society |
| 92 | Phillips, A. M. | 2016 | 'Only a click away - DTC genetics for ancestry, health, love. . .and more: A view of the business and regulatory landscape' | Appl Transl Genom |
| 93 | Ploem, et al. | 2019 | Commercieel aanbod van DNA-tests | Nederlands Juristenblad |
| 94 | Plöthner, M., et al. | 2017 | Health-Related Genetic Direct-to-Consumer Tests in the German Setting: The Available Offer and the Potential Implications for a Solidarily Financed Health-Care System | Public Health Genomics |
| 95 | Ramos, E. and Weissman, S. M. | 2018 | The dawn of consumer-directed testing | Am J Genet C Semin Med Genet |
| 96 | Roberts, J. S., et al. | 2017 | Direct-to-consumer genetic testing: User motivations, decision making, and perceived utility of results | Public Health Genomics |
| 97 | Roberts, M. C., et al. | 2019 | The FDA authorization of direct-to-consumer genetic testing for three BRCA1/2 pathogenic variants: A twitter analysis of the public's response. | JAMIA Open |
| 98 | Rollins, B. L., et al. | 2014 | Direct-to-consumer advertising of predictive genetic tests: a health belief model based examination of consumer response | Health Mark Q |
| 99 | Ryan, M.M. et al. ^a^ | 2021 | Using direct-to-consumer genetic testing results to accelerate Alzheimer disease clinical trial recruitment | Alzheimer Dis Assoc Disord |
| 100 | Salloum, R. G., et al. | 2018 | Rural-urban and racial-ethnic differences in awareness of direct-to-consumer genetic testing | BMC Public Health |
| 101 | Salm, M., et al. | 2014 | Use of genetic tests among neurologists and psychiatrists: knowledge, attitudes, behaviors, and needs for training | J Genet Couns |
| 102 | Saukko, P. | 2017 | Shifting metaphors in direct-to-consumer genetic testing: from genes as information to genes as big data | New Genetics and Society |
| 103 | Schaper et al. ^b^ | 2019 | “I would rather have it done by a doctor”—laypeople’s perceptions of direct-to-consumer genetic testing (DTC GT) and its ethical implications | Med Health Care Philos |
| 104 | Schaper, M. and Schicktanz, S. | 2018 | Medicine, market and communication: ethical considerations in regard to persuasive communication in direct-to-consumer genetic testing services | BMC Med Ethics |
| 105 | Schleit, J., et al. | 2019 | First, do no harm: direct-to-consumer genetic testing | Genet Med |
| 106 | Seward, B. | 2018 | Direct-to-Consumer Genetic Testing: Finding a Clear Path Forward | Ther Innov Regul Sci |
| 107 | Shaer, O., et al. | 2015 | Informing the Design of Direct-to-Consumer Interactive Personal Genomics Reports | J Med Internet Res |
| 108 | Sherman, K., et al. | 2015 | The effect of disease risk probability and disease type on interest in clinic-based versus direct-to-consumer genetic testing services | J Behav Med |
| 109 | Spector-Bagdady, K. | 2015 | Reconceptualizing Consent for Direct-to-Consumer Health Services | Am J Law Med |
| 110 | Spector-Bagdady, K. | 2016 | “The Google of Healthcare”: enabling the privatization of genetic bio/databanking | Ann Epidemiol |
| 111 | Spencer, E. G. and Topol, E. J. | 2019 | Direct to consumer fitness DNA testing | Clin Chem |
| 112 | Stewart, K. F. J., et al. | 2018 | Behavioural changes, sharing behaviour and psychological responses after receiving direct-to-consumer genetic test results: a systematic review and meta-analysis | J Community Genet |
| 113 | Stewart, K. F. J., et al. | 2019 | Factors Associated with Acceptability, Consideration and Intention of Uptake of Direct-To-Consumer Genetic Testing: A Survey Study | Public Health Genomics |
| 114 | Sunde, K. | 2015 | Welcome to You: A Reflection on Genetic Self-Exploration | Narrat Inq Bioeth |
| 115 | Sweeny, K., et al. | 2014 | Predictors of genetic testing decisions: a systematic review and critique of the literature | J Genet Couns |
| 116 | Tandy-Connor, S., et al. | 2018 | False-positive results released by direct-to-consumer genetic tests highlight the importance of clinical confirmation testing for appropriate patient care | Genet Med |
| 117 | Thiebes, S., et al. | 2020 | Valuable Genomes: Taxonomy and Archetypes of Business Models in Direct-to-Consumer Genetic Testing | J Med Internet Res |
| 118 | Thompson, C., et al. | 2015 | Psychiatrist attitudes towards pharmacogenetic testing, direct-to-consumer genetic testing, and integrating genetic counseling into psychiatric patient care | Psychiatry Res |
| 119 | Tiller et al. ^b^ | 2018 | Regulation of internet based Genetic testing: Challenges for Australia and other jurisdictions | Front Public Health |
| 120 | Trent, R. | 2014 | Direct-to-consumer DNA testing and the GP | Aust Fam Physician |
| 121 | Van Der Wouden, C. H., et al. | 2016 | Consumer perceptions of interactions with primary care providers after direct-to-consumer personal genomic testing | Ann Intern Med |
| 122 | Vayena, E. | 2015 | Direct-to-consumer genomics on the scales of autonomy | J Med Ethics |
| 123 | Vayena, E., et al. | 2014 | Playing a part in research? University students' attitudes to direct-to-consumer genomics | Public Health Genomics |
| 124 | VKGN | 2019 | Direct-to-consumer genetische test | *Policy report* |
| 125 | Vlahovich, N., et al. | 2017 | Ethics of genetic testing and research in sport: a position statement from the Australian Institute of Sport | Br J Sports Med |
| 126 | Vrecar, I., et al. | 2015 | Direct-to-consumer genetic testing in Slovenia: Availability, ethical dilemmas and legislation | Biochem Med (Zagreb) |
| 127 | Wasson, K., et al. | 2014 | Who are you going to call? Primary care patients' disclosure decisions regarding direct-to-consumer genetic testing | Narrat Inq Bioeth |
| 128 | Watanabe, M. et al. ^a^ | 2021 | Does direct-to-consumer personal genetic testing improve gynecological cancer screening uptake among never-screened attendees? A randomized controlled study | Int J Environ Res Public Health |
| 129 | Webborn, N., et al. | 2015 | Direct-to-consumer genetic testing for predicting sports performance and talent identification: Consensus statement | Br J Sports Med |
| 130 | Weiner, C. | 2014 | Anticipate and communicate: Ethical management of incidental and secondary findings in the clinical, research, and direct-to-consumer contexts | Am J Epidemiol |
| 131 | Wu, S., et al. | 2019 | Addressing the accuracy of direct-to-consumer genetic testing | Genet Med |
| 132 | Wynn, J. and Chung, W. K. | 2017 | 23andMe paves the way for direct-to-consumer genetic health risk tests of limited clinical utility | Ann Intern Med |
| 133 | The Lancet Oncology | 2014 | Black-box warning: Direct-to-consumer marketing | Lancet Oncology |
| ^a^ article is included in the literature update of March 2020 – June 2022 (n=7); ^b^ article was provided by experts as key article (n=2) | | | | |
