# Supplementary material for: Policy Guidance for Direct-to-Consumer Genetic Testing Services: Framework Development Study
Source: J Med Internet Res. 2024 Jul 17;26:e47389. doi: 10.2196/47389 (PMC11292153; doi:10.2196/47389)
Supplement: Multimedia Appendix 3 [file jmir_v26i1e47389_app3.docx]

| **Checklist commercial DNA-tests**  To assess DNA testing for health, disease, and lifestyle outside health care and population screening | | **Name company**  Click or tap here to enter text. | | |
| --- | --- | --- | --- | --- |
| **I Test features and background information consumer** | | *Answer* | | *Additional information* |
| 1 | What are the costs of the offered DNA-test(s)? | € Click or tap here to enter text. | | Click or tap here to enter text. |
| 2 | Which health features will be tested by the offered DNA-test(s)? | Sport  Diet  Disease risk  Pharmacogenetics  Personal features  Other, namely:  Click or tap here to enter text. | | Click or tap here to enter text. |
| 3 | Are tested features clustered per health area (sports, diet, disease risk, pharmacogenetics, personal features) or combined within a test? | Clustered  Combined  Other, namely:  Click or tap here to enter text. | | Click or tap here to enter text. |
| 4 | Can consumers opt out from receiving certain results which are analyzed by the DNA test(s) (e.g. Alzheimer’s disease, Parkinson)? | Yes  No  Unclear | Click or tap here to enter text. | |
| 5 | Will a lifestyle, sport, or diet advice be generated based on consumer results? | Yes  No  Unclear | Click or tap here to enter text. | |
| 6 | Is a referral from a health professional required to buy the DNA test(s)? | Yes  No  Unclear | Click or tap here to enter text. | |
| 7 | Is relevant (medical) background information about the consumer collected prior to the DNA test(s)?  (E.g. sex, age, lifestyle, family history, demographic background) | Yes  No  Unclear | Click or tap here to enter text. | |
| **II Technical information about the DNA test(s) and quality assurance** | | | | |
| 8 | Describe the level of information provided on the website about the type of DNA analysis that will be performed and how health risks/outcomes will be calculated (E.g. sequencing, genotyping) | Detailed  Incomplete  Missing | Click or tap here to enter text. | |
| 9 | Describe the quality of information provided on how many and which genes/variants/SNPs will be analyzed for all the health aspects | Detailed  Incomplete  Missing | Click or tap here to enter text. | |
| 10 | Does the website mention whether relevant background information about the consumer will be taken into account when computing results?  (see question 7) | Yes  No  Unclear | Click or tap here to enter text. | |
| 11 | Describe the quality of the information provided as to the laboratory conducting the analysis  (e.g. ISO-/CLIA-/CAP-certification; location; academic laboratory) | Detailed  Incomplete  Missing | Click or tap here to enter text. | |
| 12 | Is the information on the technical aspects of the DNA-test(s) and quality assurance by the companies complete and easily retrieved (question 8-12)?  Among other items, also acknowledge the format of the launch page; clear menu; the amount of click-through links; presence of a search engine.  Detailed  Almost complete  Medium  Incomplete  Inadequate  Very easy  Easy  Medium  Difficult  Very difficult | | | Click or tap here to enter text. |
| **III Transparency: privacy and data management** | | | | |
| 13 | Does the website include a privacy policy in understandable language? | Yes  No  Unclear | Click or tap here to enter text. | |
| 14 | Are terms as ‘aggregated’, ‘anonymous data’, or ‘personal data’ used and if so, are they clearly explained? | Yes  No  Unclear | Click or tap here to enter text. | |
| 15 | Describe the quality of information provided on the website regarding storage and handling of the sample after completion  (e.g. duration of storage, what will happen to the sample upon bankruptcy) | Detailed  Incomplete  Missing | Click or tap here to enter text. | |
| 16 | Describe the quality of the information provided about storage and handling of personal and genetic data (e.g. duration of storage, what will happen to the data upon bankruptcy) | Detailed  Incomplete  Missing | Click or tap here to enter text. | |
| 17 | Describe the quality of information provided about: 1) if and which data will be shared; 2) for which purposes data will be used; 3) with whom data will be shared | Detailed  Incomplete  Missing | Click or tap here to enter text. | |
| 18 | Are consumers able to withhold their personal or genetic data from being saved by the company or shared with other parties? | Yes  No  Unclear | Click or tap here to enter text. | |
| 19 | Is the information about the transparency of the company and how consumer data will be handled complete and easily retrieved (question 14-19)?  Among other things also acknowledge the format of the launch page; clear menu; the amount of click-through links; presence of a search engine.  Detailed  Almost complete  Medium  Incomplete  Inadequate  Very easy  Easy  Medium  Difficult  Very difficult | | | Click or tap here to enter text. |
| **IV Transparency: scientific evidence** | | | | |
| 20 | In case of a DNA-test on disease risk, describe the quality of the information provided about disease burden, prevalence, or incidence of tested health aspects. | Detailed  Incomplete  Missing  Not applicable | Click or tap here to enter text. | |
| 21 | Describe the evidence provided that supports methods used (e.g. reliable scientific publications) | Detailed  Incomplete  Missing | Click or tap here to enter text. | |
| 22 | Does the company collaborate with scientific partners, such as academic research groups or entitled researchers from universities (Prof., Dr.)? | Yes  No  Unclear | Click or tap here to enter text. | |
| 23 | Does the website mention that the association between genetics and health outcome may be uncertain and interpretation of the DNA-test(s) can change over time? | Yes  No  Unclear | Click or tap here to enter text. | |
| 24 | Is information about the scientific evidence of the DNA-test(s) complete and easily retrieved (question 21-24)?  Among other things, also acknowledge the format of the launch page; clear menu; the amount of click-through links; presence of a search engine.  Detailed  Almost complete  Medium  Incomplete  Inadequate  Very easy  Easy  Medium  Difficult  Very difficult | | | Click or tap here to enter text. |
| **V Information provision: interpretation, medical explanation, available actions** | | | | |
| 25 | Describe the quality of the information provided to consumers on how to interpret results | Detailed  Incomplete  Missing | Click or tap here to enter text. | |
| 26 | Does the company offer consumers the opportunity to talk to a counselor pre- or post-testing? | Yes  Against payment  No  Unclear | Click or tap here to enter text. | |
| 27 | Describe the quality of information provided about actions to be taken by consumers in case of a positive result for disease risks.  (e.g. treatment options, lifestyle changes) | Detailed  Incomplete  Missing | Click or tap here to enter text. | |
| 28 | Describe the quality and amount of information about potential effects of genes, lifestyle, environment, medication, and other factors on health outcomes | Detailed  Incomplete  Missing | Click or tap here to enter text. | |
| 29 | Does the website recommend consumers to discuss test results with a healthcare professional, counselor, or other (genetic) expert? | Yes  No  Unclear | Click or tap here to enter text. | |
| 30 | Does the website provide information on other websites that can perform additional DNA-analysis on raw consumer DNA data (so-called third-party analyzers)? | Yes  No  Unclear | Click or tap here to enter text. | |
| 31 | Is information on results’ interpretation, medical information, or available options complete and easily retrieved (question 26-31)?  Among other things, also acknowledge the format of the launch page; clear menu; the amount of click-through links; presence of a search engine.  Detailed  Almost complete  Medium  Incomplete  Inadequate  Very easy  Easy  Medium  Difficult  Very difficult | | | Click or tap here to enter text. |
| **VI Information provision: consequences of performing a DNA test** | | | | |
| 32 | Does the website mention that results can inform consumers about future health decisions and health behavior? | Yes  No  Unclear | Click or tap here to enter text. | |
| 33 | Are additional effects of performing a DNA test (beyond medical purposes) mentioned? | Yes  No  Unclear | Click or tap here to enter text. | |
| 34 | Describe the information provided about the potential risks of performing a DNA test. | Detailed  Incomplete  Missing | Click or tap here to enter text. | |
| 35 | Is it mentioned that the consequences of performing a DNA test, (potential benefits and risks), are partially unknown and are likely to change in the future? | Yes  No  Unclear | Click or tap here to enter text. | |
| 36 | Does the website mention that DNA results can have implications for taking out an insurance policy (e.g. life insurance or health insurance)? | Yes  No  Unclear | Click or tap here to enter text. | |
| 37 | Does the website mention that performing a DNA test not only affects the consumer, but also his/her family? | Yes  No  Unclear | Click or tap here to enter text. | |
| 38 | In case of detailed genetic assessment, sequencing, or markers for ancestry, does the website mention that DNA test results can shed light on (unknown) family relationships? | Yes  No  Unclear  Not applicable | Click or tap here to enter text. | |
| 39 | Does the website mention other potential effects of performing a DNA test? If yes, pleas mention which | Yes  No  Unclear | Click or tap here to enter text. | |
| 40 | Is the information on potential consequences of performing a DNA test complete and easily retrieved (question 33-40)?  Among other things, also acknowledge the format of the launch page; clear menu; the amount of click-through links; presence of a search engine.  Detailed  Almost complete  Medium  Incomplete  Inadequate  Very easy  Easy  Medium  Difficult  Very difficult | | | Click or tap here to enter text. |
| **VII Presentation of information** | | | | |
| 41 | Does the website use fear to persuade consumers to perform a DNA analysis? | Yes  No  Unclear | Click or tap here to enter text. | |
| 42 | Does the website use images or quotes from public figures or medical experts to persuade consumers to perform a DNA analysis? | Yes  No  Unclear | Click or tap here to enter text. | |
| 43 | Is there an unbalanced provision of information in which the advantages or usefulness of a DNA analysis are emphasized over their risks or disadvantages? | Yes  No  Unclear | Click or tap here to enter text. | |
| 44 | Is there a clear distinction between health outcomes/diseases: 1) for which (proven effective) treatment is available, 2) of which aspects can be partially improved, 3) for which treatment is not yet available. | Yes  No  Not applicable | Click or tap here to enter text. | |
| **VIII Informed decision making** | | | | |
| 45 | Does the website state that analyzing the DNA of others without consent is ethically irresponsible and can be punishable? | Yes  No  Unclear | Click or tap here to enter text. | |
| 46 | Does the website actively confirm that an informed decision has been made (e.g. via a pop-up)? | Yes  No  Unclear | Click or tap here to enter text. | |
| 47 | Is the information provided to consumers generally clear and understandable? | Yes  No  Unclear | Click or tap here to enter text. | |
| 48 | Considering questions 13, 20, 25, 32, 41 and 46-48, is the information needed to make an informed decision complete and easily retrieved?  Detailed  Almost complete  Medium  Incomplete  Inadequate  Very easy  Easy  Medium  Difficult  Very difficult | | | Click or tap here to enter text. |
